# INTERGENERATIONAL BENEFITS OF DISASTER PREPAREDNESS CAREGIVER INTERVENTION

**DOI:** 10.1093/geroni/igae098.3081

**Published:** 2024-12-31

**Authors:** Maria Donohoe, Morolake Adeagbo, Brittany Anderson, Aaron Seaman, Sato Ashida

**Affiliations:** University of Iowa, Iowa City, Iowa, United States; University of Iowa, Iowa City, Iowa, United States; University of Iowa, Iowa City, Iowa, United States; University of Iowa, Iowa CIty, Iowa, United States; University of Iowa, Iowa City, Iowa, United States

## Abstract

The evaluation of interventions focuses on understanding how intervention contents and components impact participant outcomes through specified mechanisms of influence. Social network literature highlights the importance of quality interactions; however, little attention has been paid to how the characteristics of interpersonal interactions may influence intervention processes and impacts. Implementation of a disaster preparedness intervention with caregivers of persons living with dementia (PLWD) began as part of a randomized controlled trial. The Disaster PrepWise-Caregiving (DPW-CG) program helps caregivers develop a household disaster and emergency management plan considering the unique family caregiving needs. Currently, DPW-CG is being delivered by trained AmeriCorps members who are college students. As part of a larger trial, a team of implementation scientists are concurrently conducting observational evaluations of the intervention delivery. The preliminary data reveals key characteristics of intergenerational interactions between the caregivers and the AmeriCorps members that influence the quality of intervention delivery. Our initial findings show that caregivers of PLWD, especially spousal caregivers who are older, looked forward to the company of AmeriCorps members and shared their life experiences and values throughout the visit. Before the commencement of intervention delivery, older caregivers strategically welcomed and eased AmeriCorps members into the process through small talk, offering food/drinks, and confidence-building compliments. This often resulted in better engagement during the intervention and positive learning experiences for the AmeriCorps members. Future intervention trials may seek to understand how characteristics of interpersonal interactions benefit program deliverers and recipients and impacts intervention effectiveness and outcomes.

